# Genetic Diversity of *Mycobacterium tuberculosis* Isolates From an Amerindian Population in Chiapas, México

**DOI:** 10.3389/fcimb.2022.875909

**Published:** 2022-07-13

**Authors:** Carmen A. Molina-Torres, Frederick D. Quinn, Jorge Castro-Garza, Anaximandro Gómez-Velasco, Jorge Ocampo-Candiani, Alied Bencomo-Alerm, Héctor Javier Sánchez-Pérez, Sergio Muñoz-Jiménez, Adrián Rendón, Afzal Ansari, Mukul Sharma, Pushpendra Singh, Lucio Vera-Cabrera

**Affiliations:** ^1^ Laboratorio Interdisciplinario de Investigación Dermatológica, Servicio de Dermatología, Hospital Universitario, Universidad Autónoma de Nuevo León, Monterrey, Mexico; ^2^ Department of Infectious Diseases, College of Veterinary Medicine, University of Georgia, Athens, GA, United States; ^3^ Centro de Investigación Biomédica del Noreste, Instituto Mexicano del Seguro Social, Monterrey, Mexico; ^4^ Departamento de Ecología Humana, Centro de Investigación y de Estudios Avanzados del Instituto Politécnico Nacional (Cinvestav), Unidad Mérida, Mérida, Mexico; ^5^ Laboratorio de Micobacterias, Programa de Prevención y Control de la Tuberculosis, región Altos de Chiapas, Instituto de Salud del Estado de Chiapas, Secretaría de Salud (SSA), San Cristóbal de Las Casas, Mexico; ^6^ Departamento de Salud, El Colegio de la Frontera Sur (ECOSUR), San Cristóbal de Las Casas, Mexico; ^7^ Centro de Investigación, Prevención y Tratamiento de Infecciones Respiratorias, Hospital Universitario, Universidad Autónoma de Nuevo León, Monterrey, Mexico; ^8^ Microbial Pathogenesis and Genomics Lab, ICMR-National Institute of Research in Tribal Health, Jabalpur, India

**Keywords:** Chiapas, tuberculosis, lineage 4, WGS, genomes, lineage 4.3.3

## Abstract

This is the first report of the genetic diversity of the *Mycobacterium tuberculosis* complex isolates found in a Mexican-Amerindian setting. In this study, we analyzed isolates collected from the Highlands region of Chiapas, Mexico, by using spoligotyping and whole-genome sequencing analyses. Seventy-three *M. tuberculosis* isolates were analyzed initially by spoligotyping; no new spoligotypes were identified. Nineteen percent of the isolates were identified as SIT53 (T1) (n = 14), followed by SIT42 (14%, n = 10, LAM9) and SIT119 (11%; n = 8, X1). SIT53, SIT42, and orphan isolates (16.4%, n = 12) constituted about 50% of the isolates studied and were subjected to whole-genome sequencing (WGS) analysis. Most SIT53 (10/12) isolates belonged to the Euro-American sub-lineage 4.8. Most SIT42 isolates (4/7) as .well as most orphan isolates (5/8) belonged to the lineage 4.3.3 LAM group. By comparing the single-nucleotide polymorphism (SNP) patterns of the SIT53 isolates, we found one clone (<7 SNPs) and four clustered isolates (<15 SNPs). In isolates from the SIT42 and orphan groups, we did not find any clones or clusters. This work demonstrates the success of sub-lineage 4.8 to predominate in Mexico and confirms the dominion of sub-lineage 4.3.3 in Central and South America.

## Introduction

Prior to the COVID-19 pandemic, tuberculosis (TB) was the world leading cause of human death due to a single infectious agent. In México, TB is a serious public health issue with contrasting prevalence among the different geographic regions (Sánchez-Barriga, 2015). Chiapas, a Southern state located in the Mexico-Guatemala border, is one of the poorer states and has one of the highest TB morbidity and mortality rates in the country (Sánchez-Barriga, 2015). Factors that contribute to this situation include poverty, low educational levels, inadequate health service coverage, failure to comply the strict directly observed treatment (DOT) for TB, and a growing epidemic of resistance to first-line drugs (Sánchez-Pérez et al., 2001; Nájera-Ortiz et al., 2008; Sánchez-Pérez et al., 2010; Nájera-Ortiz et al., 2012).

Overall, the Mexican population comprised divergent ancestry such as a mixture of the original descendants of American indigenous populations, the Amerindians, and Europeans, mostly of Spanish heritage (Moreno-Estrada et al., 2014), but the regional ethnic composition may vary greatly. For example, in Nuevo León, a state located in the North of México, pure Amerindians account for only 1% of the population, while in Chiapas, the Amerindian population may constitute up to 28%, mainly from Mayans descendants (Moreno-Estrada et al., 2014).

Little is known about the genetic diversity of the *M. tuberculosis* complex (MTBC) isolates circulating in Mexican-Amerindian settlements. The presence of MTBC species in America prior to the arrival of Europeans (pre-Columbian) has been a source of controversy. It has been proposed that the presence of *M. pinnipedii* in some South American mummies could be explained by the migration of sea lions from the Cape of Good Hope in Africa, first to South America and later to Central and North America (Bos et al., 2014). If such a hypothetical scenario is correct, the MTBC isolates found in America should have different spoligotypes than those typically found in European descendants. In order to identify the predominant genetic patterns of MTBC isolates, we conducted a study in an Amerindian rural population settled in the mountains of the Highlands region of Chiapas using spoligotyping and whole-genome sequencing (WGS) analyses.

## Materials and Methods

### *M. tuberculosis* Isolates

Human clinical isolates were obtained from the Laboratorio de Micobacterias, Programa de Prevención y Control de la Tuberculosis, Instituto de Salud del Estado de Chiapas in 2015 and 2016 (n = 73), including patients from indigenous rural communities in the Highlands region (Los Altos). Only one patient was HIV positive. Data on drug susceptibility were not available, and the patients received empiric treatment based on a positive acid-fast sputum smear. The specimens were decontaminated by the NALC-NaOH method and cultured on a Lowenstein-Jensen medium.

Prior to the start of the study, ethical approval was obtained from the Ecosur Research Ethics Committee (CEI-O-068/14) and from the Ministry of Health for Chiapas (5003/00063) under the project “*Mycobacterium tuberculosis* spoligotypes from clinical isolates from Chiapas, Mexico”. The microbiological records and basic demographic information for patients were anonymized and de-identified prior to analysis. Informed consent for participation was obtained from patients in a written form, and sputum samples were used only for the purpose of this study.

### Spoligotyping

DNA was isolated using the method of Van Soolingen et al. (1991). In brief, colonies from LJ slants were collected in a microcentrifuge tube containing 500 μl of TE buffer (0.01 M Tris–HCl, 0.001 M EDTA [pH 8.0]) and then heated at 80°C for 30 min to kill the cells. Lysozyme was added to a final concentration of 1 mg/ml, and the tube was incubated for 1 h at 37°C. Seventy microliters of 10% sodium dodecyl sulfate (SDS) and 6 μl of proteinase K (at a 10 mg/ml concentration) were added, and the mixture was incubated for 10 min at 65°C. Eighty microliters of N-cetyl-N,N,N-trimethyl ammonium bromide was added. The tubes were vortexed briefly and incubated for 10 min at 65°C. An equal volume of chloroform-isoamyl alcohol (24:1, vol/vol) was added, and the mixture was vortexed for 10 s. After centrifugation for 5 min, 0.6 volume of isopropanol was added to the supernatant to precipitate the DNA. After 30 min at -20°C and centrifugation for 15 min, the pellet was washed once with 70% ethanol and the air-dried pellet was redissolved in 50 μl of 0.1× TE buffer (0.001 M Tris–HCI, 0.0001 M EDTA [pH 8.0]). Spoligotyping was carried out using a standard technique previously described (Driscoll, 2009). The spoligotypes obtained were entered into the SITVITWEB database (Pasteur Institute of Guadeloupe) in a binary format (http://www.pasteur-guadeloupe.fr:8081/SITVIT_ONLINE/). Restriction fragment length polymorphism (RFLP) analysis for IS6110 was performed by Southern blotting with labeled IS6110 DNA as previously described (Van Embden et al., 1993).

### Whole-Genome Sequencing

Not all the isolates were subjected to genome sequencing, since some isolates were lost. We analyzed a total of 27 (SIT53, n = 12; SIT42, n = 7 and eight orphans) isolates. Genomic DNA isolation and preparation was done as described for RFLP above. DNA libraries were prepared using the Nextera XT DNA Library Preparation Kit (Illumina, San Diego, CA, USA) and checked for quality using a fragment analyzer (Advanced Analytical Technologies, Inc. (AATI), Santa Clara, CA, USA) before sequencing using a MiSeq sequencer (Illumina). Average coverage ranged from 99.19 to 99.86 and depth from 69.7x to 210.98x. The Whole Genome Shotgun project has been deposited at DDBJ/ENA/GenBank under the following accession numbers: *M. tuberculosis* LIID-CH2 (JAIFJQ000000000), *M. tuberculosis* LIID-CH3 (JAIFJP000000000), *M. tuberculosis* LIID-CH6 (JAIFJO000000000), *M. tuberculosis* LIID-CH14 (JAIFJN000000000), *M. tuberculosis* LIID-CH15 (JAIFJM000000000), *M. tuberculosis* LIID-CH17 (JAIFJL000000000), *M. tuberculosis* LIID-CH18 (JAIFJK000000000), *M. tuberculosis* LIID-CH19 (JAIFJJ000000000), *M. tuberculosis* LIID-CH21 (JAIFJI000000000), *M. tuberculosis* LIID-CH24 (JAIFJH000000000), *M. tuberculosis* LIID-CH28 (JAIFJG000000000), *M. tuberculosis* LIID-CH30 (JAIFJF000000000), *M. tuberculosis* LIID-CH31 (JAIFJE000000000), *M. tuberculosis* LIID-CH33 (JAIFJD000000000), *M. tuberculosis* LIID-CH40 (JAIFJC000000000), *M. tuberculosis* LIID-CH42 (JAIFJB000000000), *M. tuberculosis* LIID-CH53 (JAIFJA000000000), *M. tuberculosis* LIID-CH60 (JAIFJZ000000000), *M. tuberculosis* LIID-CH68 (JAIFJY000000000), *M. tuberculosis* LIID-CH70 (JAIFJX000000000), *M. tuberculosis* LIID-CH76 (JAIFJW000000000), *M. tuberculosis* LIID-CH93 (JAIFJV000000000), *M. tuberculosis* LIID-CH109 (JAIFJU000000000), and *M. tuberculosis* LIID-CH117 (JAIFJT000000000). All these sequences belong to BioProject PRJNA751891.

The raw fastq files were analyzed with the Phylo-Resistance Search Engine (PhyResSe) (Feuerriegel et al., 2015). To start the analysis, all reads from each isolate were mapped to the *M. tuberculosis* H37Rv reference strain (GenBank accession no. NC_000962.3) with BWA-MEM using the Sequencher suite 5.4.6 (Gene Codes, Ann Arbor, MI). The scaffolds in fasta format were analyzed using PhyResSe. The software produces a phylogenetic tree, a list of genetic variants including insertions, deletions, and single-nucleotide polymorphisms (SNPs), and the lineage resulted of each isolate. It also identifies well-described mutations that are known to confer antibiotic resistance and by using a combination of SNPs that are markers for specific genotypes, as well as mutations that are more deeply rooted in the MTBC diversity and therefore characterize a larger set of strains (e.g., the Euro-American lineage). The phylogenetic trees were prepared using the genome sequences in FASTA format with the Type (Strain) Genome Server (TYGS) (Meier-Kolthoff and Göker, 2019) and uploaded. The TYGS is a free bioinformatics platform available under https://tygs.dsmz.de, for a whole-genome-based taxonomic analysis. The resulting intergenomic distances were used to infer a balanced minimum evolution tree with branch support *via* FASTME 2.1.6.1 including SPR postprocessing. Branch support was inferred from 100 pseudo-bootstrap replicates each. The trees were rooted at the midpoint and visualized with PhyD3 and iTOL tools.

## Results

### Spoligotypes

A total of 73 *M. tuberculosis* isolates were analyzed, from which 58.9% (n = 43) of samples were from men and 41% (n = 30) were from women. In **Table 1**, we summarize their demographic and clinical data. Descriptions of the 29-spoligotype patterns were obtained from the SITVITWEB database and classified into seven clades as shown in **Table 2**. The most frequently identified subclades were SIT53 (n = 14, 19.2%), SIT42 (n = 10, 13.7%), SIT119 (n = 8, 11%), and SIT106 (n = 4, 5.5%) from the T1, LAM9, X1, and H1 spoligotypes, respectively. The T lineage was found to be predominant with 21 isolates, followed by the LAM lineage with 20 isolates. Twelve isolates were not reported in the data base.

**Table 1:** Demographic and clinical data of the study participants.

Isolate	Age	Sex	Municipality	SIT	AFB	Diagnostic date	TB Infection	Sample	Occupation	Schooling	Ethnic group	Comorbidities			
												HIV	D	M	A
LIID-CH1	54	M	Chamula	42	+++	24/04/2014	P	S			Tzotzil				
LIID-CH2	45	M	Chalchihuitan	42	+++	12/05/2014	P	S	F	NS	Tzotzil			X	
LIID-CH3	64	F	Teopisca	53	+++	29/05/2014	P	S					X		
LIID-CH4	35	F	Tenejapa	93	+++	02/06/2014	P	S			Tzeltal				
LIID-CH6	46	F	San Juan Cancuc	O	+++	17/05/2014	P	S	HW	NS	Tzeltal				
LIID-CH8	46	F	Zinacantan	50	+++	07/07/2014	P	S	HW	NS	Tzotzil				
LIID-CH9	37	M	Chamula	522	+++	27/06/2014	P	S	F	NS	Tzotzil			X	
LIID-CH10	49	M	SCLC	450	+++	13/08/2014	P	S							X
LIID-CH11	14	F	Tenejapa	119	+++	16/08/2014	G	GJ			Tzeltal				
LIID-CH12	75	M	Zinacantan	42	+	21/08/2014	P	S	F	NS	Tzotzil				
LIID-CH13	29	F	SCLC	O	+	22/02/2014	P	S	E	E				X	
LIID-CH14	17	F	SCLC	53	+++	11/09/2014	P	S	S	HS					
LIID-CH15	49	M	Zinacantan	O	+++	17/09/2014	P	S			Tzotzil		X		
LIID-CH17	78	M	Zinacantan	42	+++	19/09/2014	P	S			Tzotzil				
LIID-CH18	18	M	Chamula	42	+++	21/09/2014	P	S			Tzotzil		X		
LIID-CH19	45	F	Chamula	53	+++	03/09/2014	P	S			Tzotzil				
LIID-CH21	31	M	Larrainzar	42	+++	04/09/2014	P	S	F	E	Tzotzil			X	
LIID-CH22	22	M	Chalchihuitan	1347	+++	08/09/2014	P	S	F	NS	Tzotzil				
LIID-CH23	57	F	SCLC	47	+++	01/10/2014	P	S	HW	IE			X		
LIID-CH24	25	M	Chalchihuitan	O	+++	29/09/2014	P	S	F	NS	Tzotzil			X	
LIID-CH-27	34	F	Tenejapa	33	+++	03/10/2014	P	S			Tzeltal				
LIID-CH-28	34	M	Chamula	O	+++	14/10/2014	P	S	F	IE	Tzotzil			X	
LIID-CH-29	23	F	SCLC	119	+++	18/10/2014	P	S							
LIID-CH-30	19	M	Teopisca	53	+++	02/11/2014	P	S	C	HS					
LIID-CH-31	23	F	SCLC	O	+										
LIID-CH-32	18	F	Simojovel	119	+++	29/10/2014	P	S	HW	E					
LIID-CH-33	33	M	Oxchuc	53	+++	29/10/2014	P	S	F	NS	Tzeltal			X	
LIID-CH-34	19	M	Teopisca	53	+++	29/10/2014	P								
LIID-CH-35	30	F	Chalchihuitan	119	+++	03/11/2014	P	S	HW	E	Tzotzil				
LIID-CH37	28	M	Zinacantan	2285	+	05/11/2014	P	S	F	NS	Tzotzil				
LIID-CH40	72	M	San Juan Cancuc (Ocosingo)	53	+++	16/11/2014	P	S	F	IE	Tzeltal				
LIID-CH42	47	M	Teopisca	O	+	20/01/2015	P	S	F	NS				X	
LIID-CH43	32	M	Aldama	O	+++	22/01/2015	P	S	F	MI	Tzotzil			X	
LIID-CH44	31	F	SCLC	207	+++	31/01/2015	P	S	T	M				X	
LIID-CH46	38	M	Zinacantan	O	++	15/02/2015	P	S			Tzotzil				
LIID-CH47	42	M	Zinacantan	93	+++	13/02/2015	P	S			Tzotzil				
LIID-CH50	50	M	Pujiltic	60	+	17/02/2015	P	S	F	IE					
LIID-CH51	32	M	San Juan Cancuc	O	+++	25/02/2015	P	S	F	E	Tzeltal				
LIID-CH52	38	M	Zinacantan	119	+++	25/02/2015	P	S	D	IE	Tzotzil		X		
LIID-CH53	46	F	Larrainzar	42	+++	05/03/2015	P	S	HW	IE	Tzotzil		X		
LIID-CH54	84	M	SCLC	119	+++	10/04/2015	P	S							
LIID-CH60	18	F	SCLC	53	+++	01/06/2015	P	S	HW	MI					
LIID-CH61	22	M	Oxchuc	240	+++	22/05/2015	P	S			Tzeltal				
LIID-CH62	20	F	Chamula	60	+++	11/06/2015	P	S	HW	IE	Tzotzil			X	
LIID-CH63	7	F	Chalchihuitan	119	+++	06/08/2015	P	GJ		NS	Tzotzil			X	
LIID-CH66	25	F	Chenalho	1347	+++	05/08/2015	P	S	HW	NS	Tzotzil			X	
LIID-CH67	35	F	SCLC	Beijing	+++	25/08/2015	P	S	T	M			X		
LIID-CH68	19	F	Ocosingo	53	+	27/08/2015	P	S							
LIID-CH69	23	F	Chamula	118	+++	21/08/2015	P	S	HW	NS	Tzotzil			X	
LIID-CH70	28	M	Chenalho	53	+++	29/08/2015	P	S	C	E	Tzotzil	X			
LIID-CH76	70	M	Oxchuc	42	+	03/11/2015	P	S	F	IE	Tzeltal				
LIID-CH77	65	M	Chenalho	53	+++	13/01/2016	P	S			Tzotzil				
LIID-CH79	50	M	Tenejapa	106	+++	21/01/2016	P	S	F	E	Tzeltal		X		
LIID-CH82	71	F	SCLC	53	No	05/02/2016	P								
LIID-CH86	45	F	SCLC	O	+	08/08/2016	P	S	HWs	IE			X		
LIID-CH89	31	M	El Bosque	1356	+++	15/08/2016	P	S			Tzotzil				
LIID-CH90	38	F	Huixtan	106	+++	19/08/2016	P	S	HW	IE	Tzotzil			X	
LIID-CH93	45	M	Chilon	O	+++	02/09/2016	P	S			Tzeltal				
LIID-CH96	25	F	Tuxtla Gutierrez	273	+++	05/09/2016	P	S							
LIID-CH98	72	M	Chalchihuitan	60	+++	07/09/2016	P	S	F	NS	Tzotzil				
LIID-CH101	47	M	Larrainzar	291	+++	07/09/2016	P	S			Tzotzil				
LIID-CH102	66	F	Larrainzar	47	++	09/09/2016	P	S			Tzotzil				
LIID-CH103	34	M	Zinacantan	106	+++	12/09/2016	P	S	D	IE	Tzotzil		X		
LIID-CH104	62	M	Oxchuc	452	+++	14/09/2016	P	S	F	IE	Tzeltal				
LIID-CH106	61	F	El Pinar	53	++	06/10/2016	P	S	HW	NS	Tzotzil		X		
LIID-CH108	71	M	Simojovel	42	+++	20/12/2016	P	S							
LIID-CH110	19	M	Tenejapa	42	+++	20/02/2017	P	S	F	E	Tzeltal				
LIID-CH112	41	M	Zinacantan	106	++	02/03/2017	P	S	F	NS	Tzotzil				
LIID-CH114	62	M	Teopisca	119	+++	01/03/2017	P	S	F	NS			X		
LIID-CH115	34	M	Chamula	O	+++	30/03/2017	P	S			Tzotzil				
LIID-CH117	71	M	San Juan Cancuc	53	+++	03/04/2017	P	S	F	NS	Tzeltal				
LIID-CH118	18	M	Chamula	273	++	03/04/2017	P	S	T	MI	Tzotzil				X
LIID-CH119	60	M	Amatenango del Valle	33	+++	10/04/2017	P	S			Tzeltal				

O, orphan; P, pulmonary; G, gastric; S, sputum; GJ, gastric juice; F, farmer; HW, housewife; E, employee; T, technician; C, craftsman; NS, non schooling; E, elementary; HS, high school; IE, incomplete elementary; MS, middle school; M, master of sciences; D, diabetes, M, malnutrition; A, alcoholism.The plus signs represent the intensity of the bacterial load in the sputum sample stained with Ziehl-Nielsen. From 1+ representing less than 9 bacilli per 100 fields observed, to 4+, representing more than 10 bacilli in 20 fields observed. (In: Manual for the bacteriological diagnosis of tuberculosis. Part 4: manual of external Quality assessment procedures of bacteriological methods applied to diagnosis and Treatment monitoring of tuberculosis / Program “Strengthening the Tuberculosis Laboratories. Networks in the Region of the Americas” - Lima: ORAS - CONHU; 2019).

**Table 2:** Families, spoligotype patterns, octal codes and frequency of the 73 MTBC genotypes identified.

	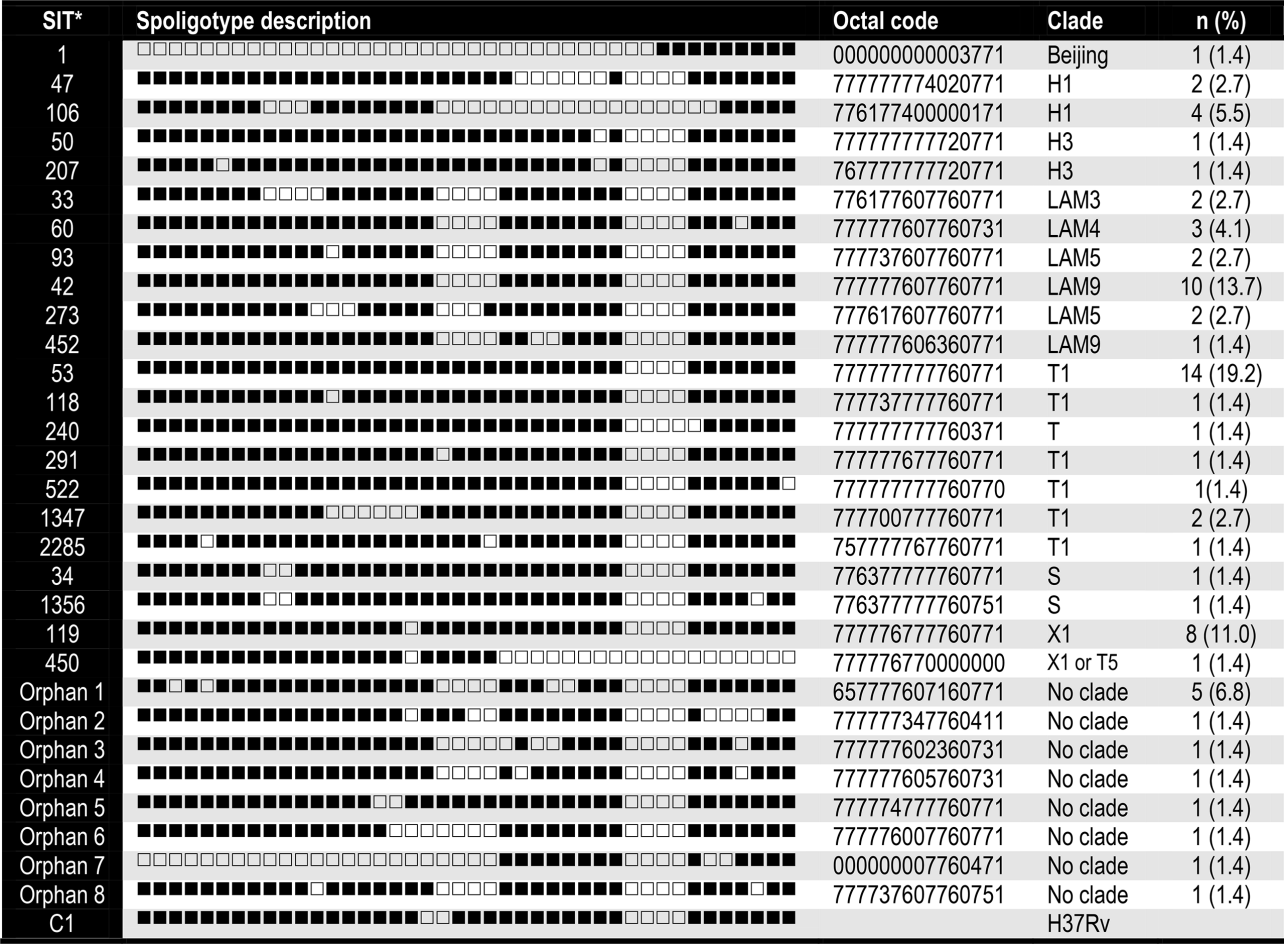	

*SIT: Spoligo international type numbers from the SpolDB4 database.

### WGS Analysis of SIT53 Isolates

**Figure 1:**
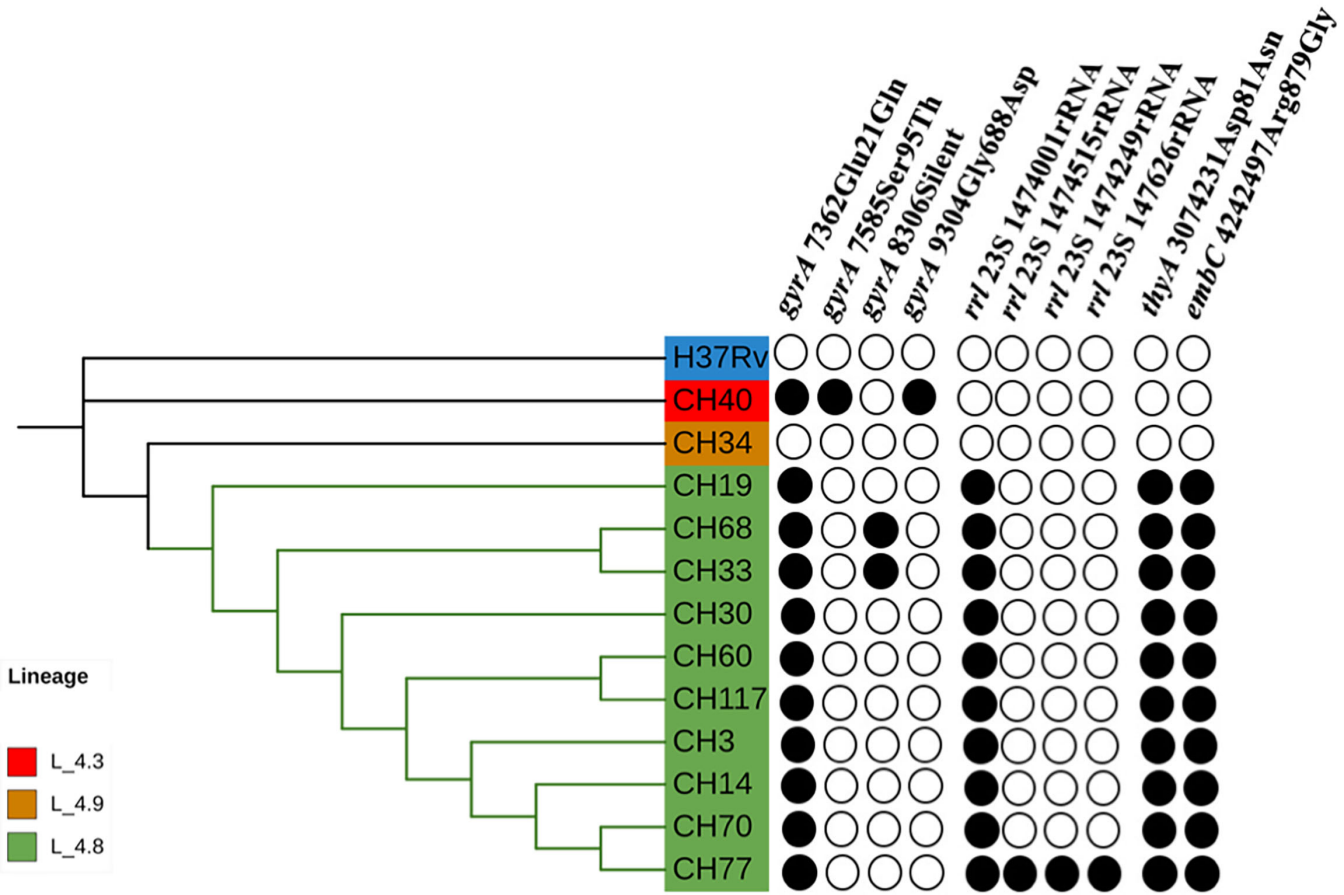
Phylogenetic tree showing the relationships among isolates belonging to SIT53. Mutation patterns observed in *gyrA*, rRNA operon (*rrl*), *thyA* (para-aminosalicylic acid, PAS), and *embC* (ethambutol) are shown as filled circles.

**Figure 2:**
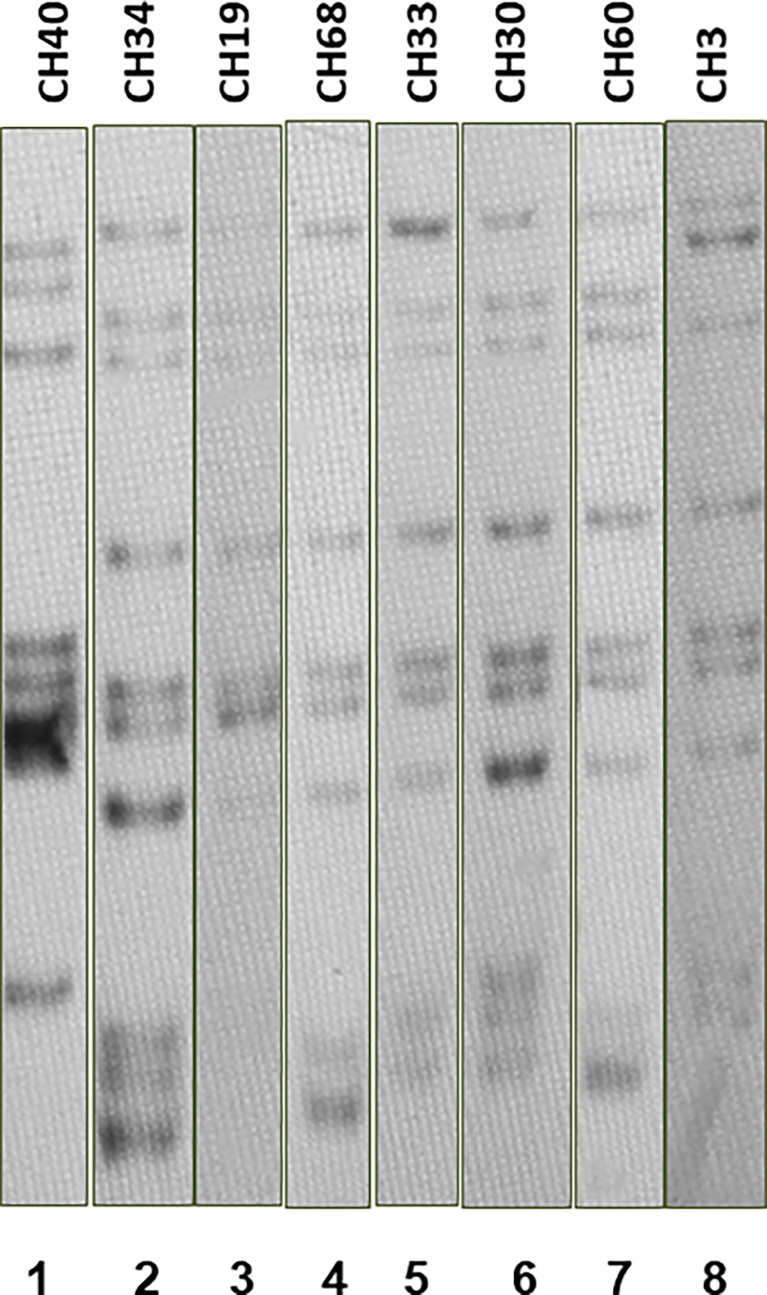
IS*6110* RFLP patterns for MTB SIT53 isolates belonging to lineage 4.8 (CH19 to CH3). All 4.8 lineage isolates (lanes 3 to 8) presented the same IS*6110* pattern (except CH40), as well as CH34 (lane 2) which belongs to lineage 4.0.

Most SIT53 isolates belong to sub-lineage 4.8, with exceptions of CH40 (lineage 4.3.4.1) and CH34 (lineage 4.9) (**Figure 1**); in the phylogenetic tree, these two isolates are close to the outgroup, MTBH37Rv (**Figure 1**). When analyzing the presence of mutations in drug-resistance-associated genes such as *gyrA* (quinolones), *rrl* (streptomycin), *thyA* (para-aminosalicylic acid; PAS), and *embA* (ethambutol), a clear difference was observed between CH34 and CH40 and the rest of the isolates. CH34 showed no mutations in these genes, whereas CH40 presented a completely different pattern (**Figure 1**). However, none of these changes presented in **Figure 1** have been found associated with phenotypic resistance according to the TB profiler list of SNPs (Phelan et al., 2019). When analyzing their IS6110 RFLP patterns, SIT53 4.8 isolates share a similar seven-band pattern (**Figure 2**), except for the CH40 isolate.

**Figure 3:**
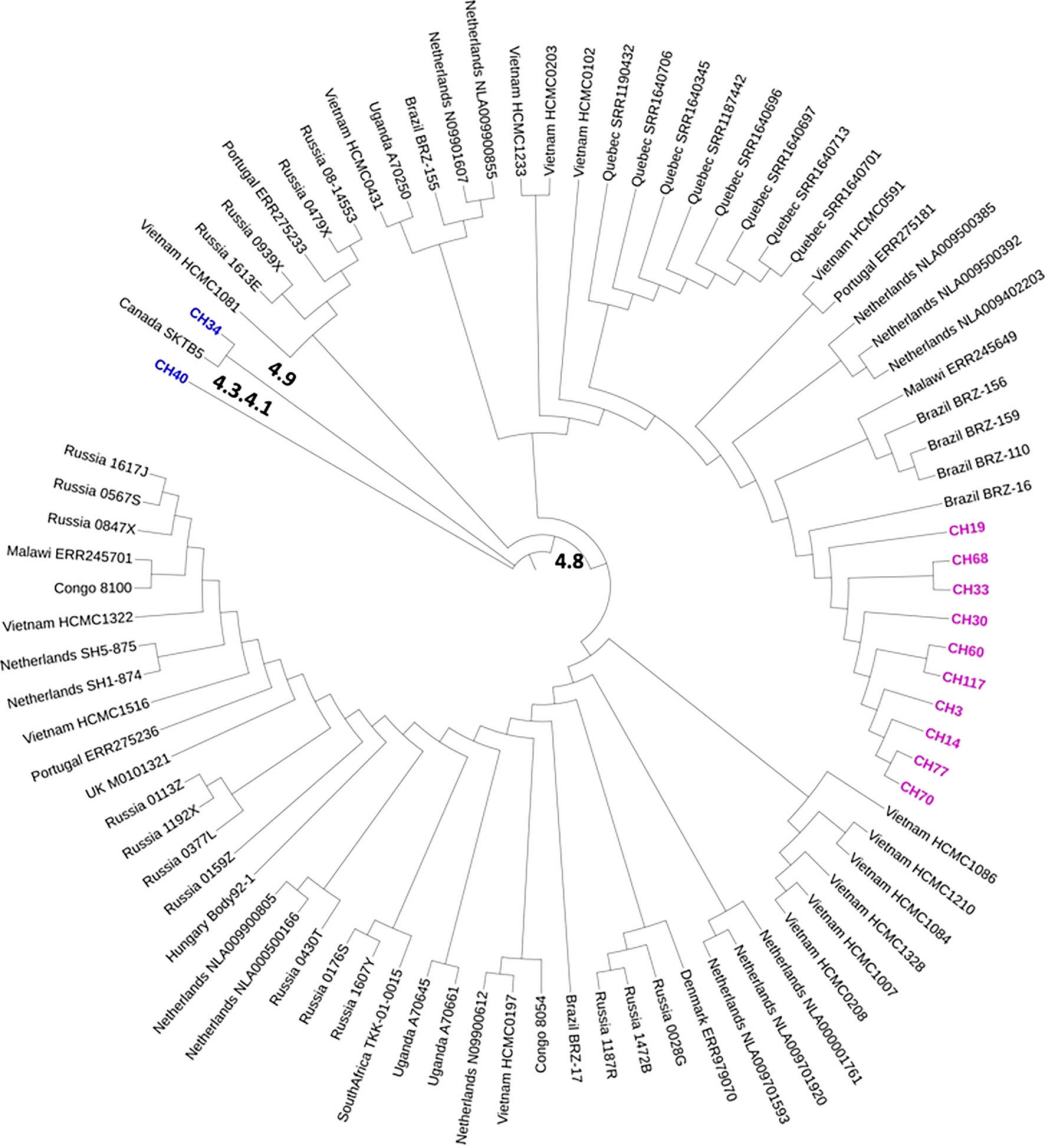
Comparative phylogeography of a 4.8 global dataset with the genomes of the SIT43 MTB isolates from Los Altos de Chiapas. In the map, we show in pink the cluster formed by the 4.8 isolates. In blue, other SIT53 isolates belonging to different 4.0 lineages are represented. The genome sequences were obtained from those published and deposited in GenBank by Brynildsrud et al. (2018).

Genomes of the SIT53 isolates were compared to *M. tuberculosis* lineage 4 genomes (**Figure 3**) from around the world (Brynildsrud et al., 2018). It was found that all the Chiapas 4.8 lineage isolates clustered together had the highest similarity with Brazilian 4.8 sub-lineage isolates followed by Netherlanders and Quebecois (**Figure 3**).

### WGS Analysis of SIT42 and Orphan Spoligotypes

**Figure 4:**
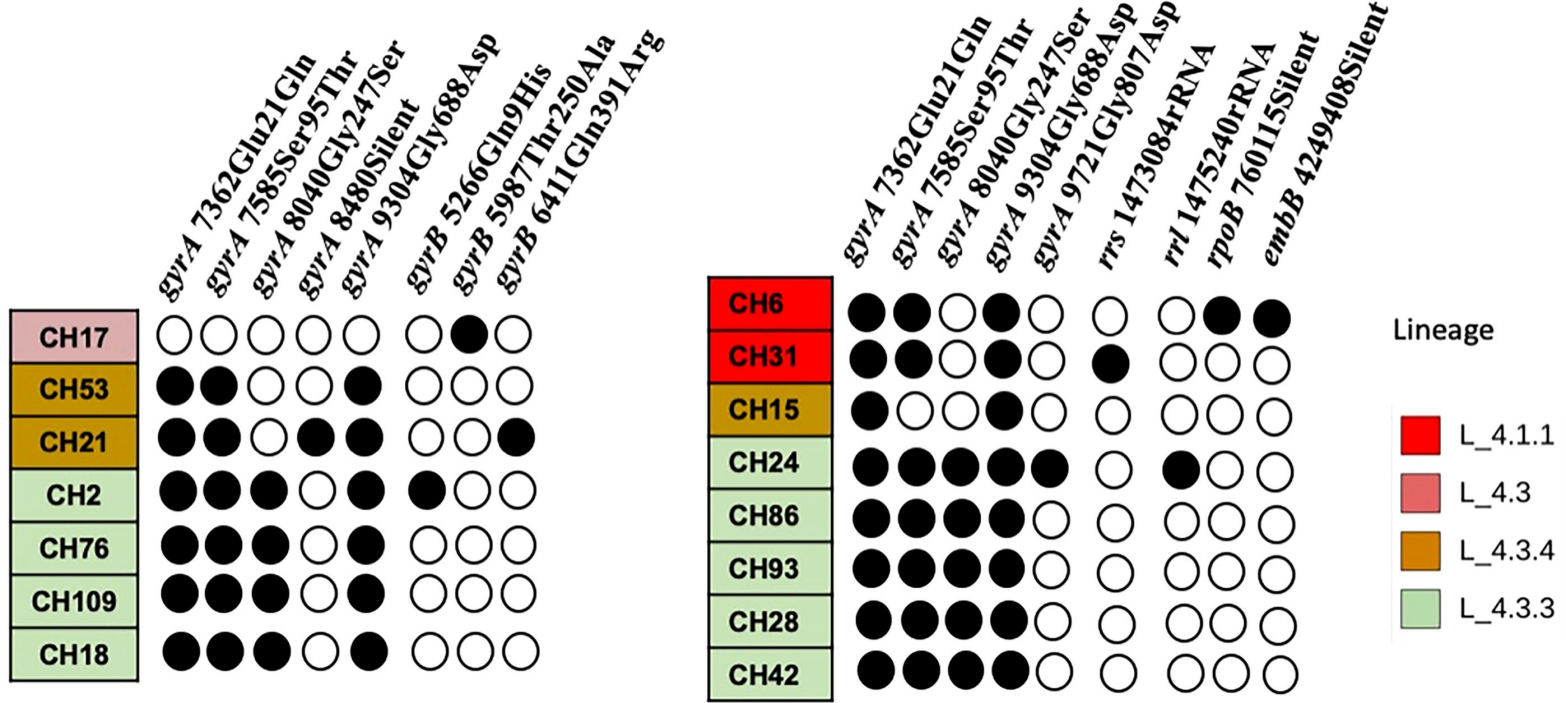
SNP pattern of SIT42 and orphan isolates of genes involved in antituberculosis drug resistance. Mutations observed in DNA gyrase subunit A (*gyrA*), B (*gyrB*), the *rrs* and 23S rRNA (*rrl*) subunits, the beta subunit of RNA polymerase (*rpoB*), and the arabinosyltransferase subunit B (*embB*) are shown in black circles. The isolates are ordered according to their phylogenetic relationships.

When comparing the SNP patterns of SIT42 isolates, they differ between 75 (CH109) and 194 (CH17) SNPs. None of them clustered (>12 SNPs) or were considered to belong to an outbreak (<5 SNPs) (not shown), although four out of seven shared a common deletion pattern in the *gyrA* gene at nucleotides 7362, 7585, 8040 and 9304 and belong to lineage 4.3.3 (**Figure 4**, left). The rest of SIT42 isolates belong to lineages 4.3.4 and 4.3.

Five out of eight orphan isolates belonged also to lineage 4.3.3 and also share the same four SNPs in the *gyrA* gene (**Figure 4**, right). Isolate CH15 belongs to LAM 4.3.4, and CH31 and CH6 belong to Ghana, Haarlem, X-type, 4.1.1, respectively, and presented a different pattern of mutations in antimicrobial resistance-associated genes. Interestingly, most orphan isolates belonging to lineage 4.3.3 have lost spacers 21 to 24 and 33 to 36 (not shown) of their spoligotype pattern. Although isolate CH86 belongs to 4.3.3, it presents a completely different spoligotype pattern. Orphan isolates differ from the *M. tuberculosis* H37Rv genome sequence at several positions (>714 SNPs) and also differ among themselves by 78 to 499 SNPs (not shown).

**Figure 5:**
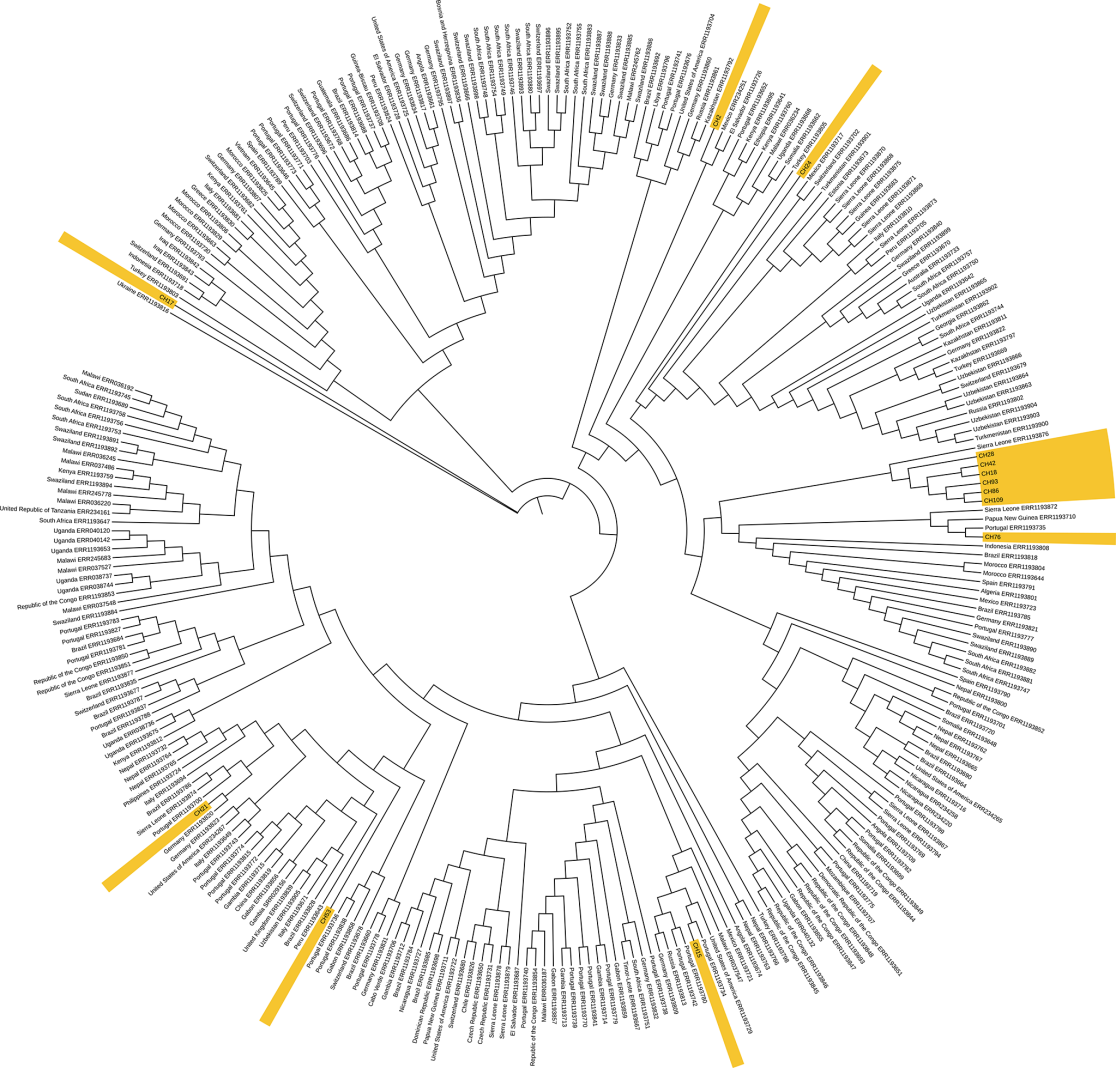
Genome-based phylogeny map of lineage 4.3 isolates from Chiapas, belonging to SIT42 and orphan SITs, and 293 genomes of 4.3 sub-lineage isolates from the world. In yellow, we show those from our study.

When comparing with other isolates from the 4.3 lineage from the world, the two SIT42 (CH109, and CH 18) and 4 orphan (CH28, CH42, CH93, and CH86) formed a cluster close to 4.3 isolates from Sierra Leona (**Figure 5**). Besides this clade, CH76 (a SIT42 isolate) clusters with isolates from Sierra Leone and Portugal. These Chiapas isolates belong to the group sharing mutations at the *gyrA* gene at nucleotides 7362, 7585, 8040, and 9304. CH2 (SIT42) and CH24 (ORF) were other 4.3.3 isolates that shared the *gyrA* mutations but clustered close to isolates of Mexican origin. CH21 (4.3.4, SIT42), CH53 (4.3.4, SIT42), and CH15 (4.3.4, ORF) were located close to isolates from Portugal. CH17 (4.3, SIT42) was located far from the other isolates with a Ukraine isolate.

**Figure 6:**
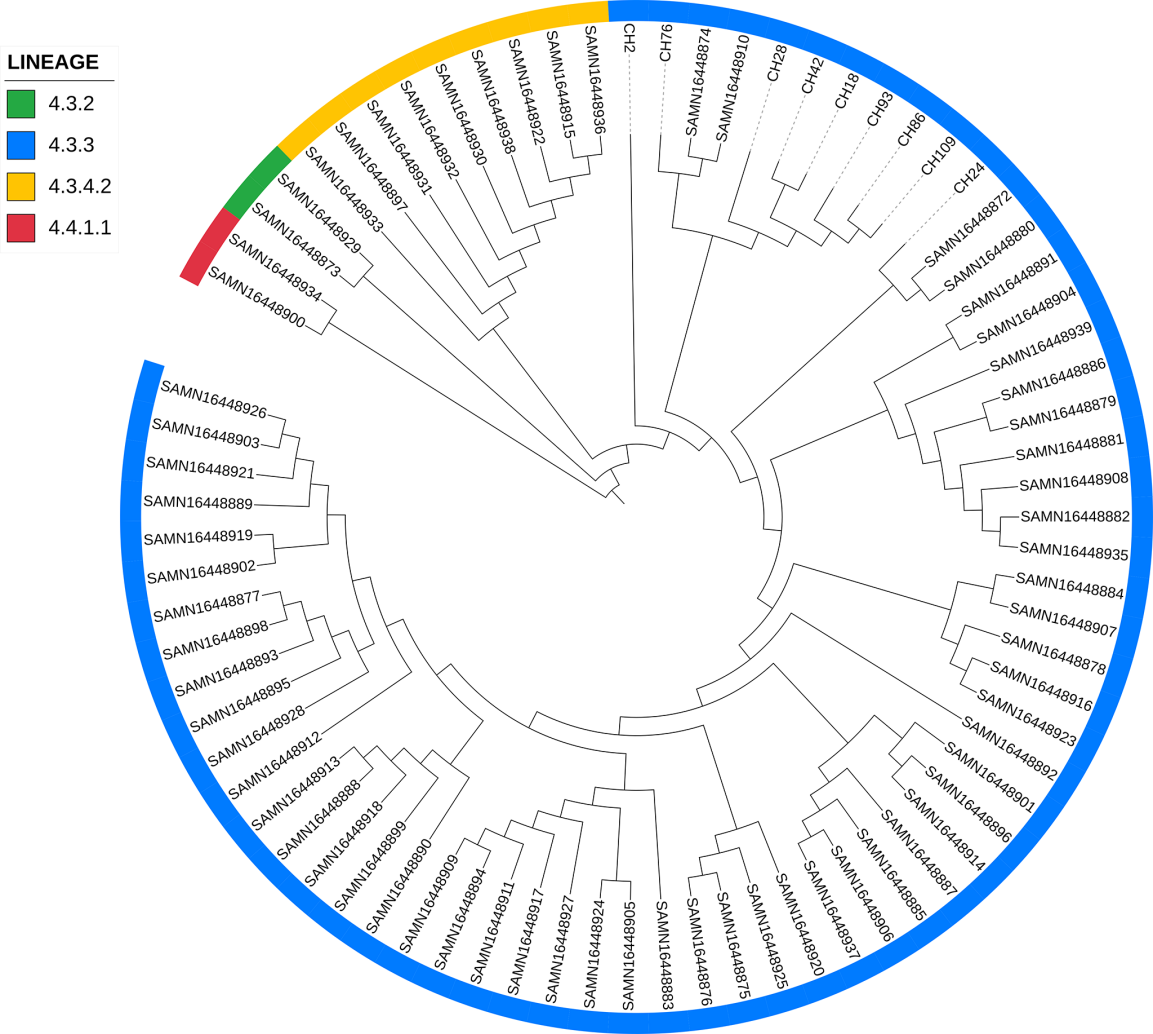
Comparison of lineage 4.3.3 isolates from Chiapas to genomes of *M. tuberculosis* isolates obtained from Amazonic populations. Lineages of every isolate is marked with different colors in the periphery.

When comparing the genomes of SIT42 or orphan isolates belonging to lineage 4.3.3 with the genomes of MTB isolates from Amazonic Amerindian populations of Brazil (Hadi et al., 2021), the Mexican genomes, except for CH2, clustered together, with two Amazonic isolates (SAMN16448874 and SAMN16448910) intercalated among them (**Figure 6**).

## Discussion

Chiapas is a Southern Mexican state with approximately 7.3% of Mexico’s indigenous population (INEGI, 2015). The state was a part of a large region dominated by the Mayans during the Classic period (A.D. 250 to 900), and people currently living in this region are predominantly their descendants. The most numerous of these Mayan groups include the Tzeltal, Tzotzil, Ch’ol, Zoque, Tojolabal, Lacandon, and Mam (INEGI, 2004). Most communities are found in the municipalities of the Centro, Altos, Norte, and Selva regions, with many considered to be 100% indigenous (INEGI, 2015).

To date, several studies have described *M. tuberculosis* genetic diversity around Mexico, but not in Amerindian populations. Previous studies have shown SIT53 (T1) to be the most predominant subclade found in Monterrey (24%), Guadalajara (25%), and San Luis Potosí (28.6%) (Molina-Torres et al., 2010; López-Rocha et al., 2013; Flores-Treviño et al., 2015). In our results in Chiapas, SIT53 represented 19.3% of the total isolates examined.

Interestingly, SIT53 isolates from Chiapas presented seven IS6110 bands; in our previous study in Monterrey, SIT53 strains showed four bands or less (Molina-Torres et al., 2010). In Mexico City (Zenteno-Cuevas et al., 2015), isolates corresponding to SIT53 were multi-banded strains but the band pattern was not found to be similar to the Chiapas profile. Clusters of strains with the same spoligotype can present different IS6110 profiles because it has been estimated that the rate of change in the DR region is slower than that of IS6110 (Niemann et al., 1999). IS6110 is a genetic clock that shows recent changes in the genome (Niemann et al., 1999; Warren et al., 2002). In our study, the identical IS6110 pattern between SIT53 isolates indicates a recent transmission (Cave et al., 2005). Different and assorted IS6110 patterns among SIT53 isolates from the entire country indicate a common ancestor probably long time ago when lineage 4 was introduced by Europeans into the country, about 1800 A.D. (Brynildsrud et al., 2018). A wider WGS analysis of SIT53 isolates from Mexico will help to trace the evolution of these strains over the 500 years since the arrival of these *M. tuberculosis* isolates, and how they have been adapted to the Mexican population.

Lineage 4 *M. tuberculosis* strains are originally from Europe; from there, they migrated to Vietnam in the 13th century (Brynildsrud et al., 2018), to the Republic of Congo, Africa, in the 15th century, and then to Uganda and Malawi in the late 17th century. The three earliest migration events to America were inferred to have occurred between 1466 and 1593 to South America and between 1566 and 1658 to North America (Brynildsrud et al., 2018). Most of our SIT 53 isolates belonged to lineage 4.8, which is not commonly found in America, where 4.3.3 dominates (Hadi et al., 2021). This might be explained by groups of people from Spain, where Lineage 4 is predominant (Madrazo Moya et al., 2021), or Portugal who migrated to Mexico or South America carrying different sub-lineages, or to the adaptation through the years to the local hosts, as has been observed in *in vivo* experiments (Reiling et al., 2013). When comparing our 4.8 isolates with other isolates of the world (Stucki et al., 2016), we observed a closer relationship with strains from Brazil, strengthening this possibility. Interestingly, we found a lineage 4.9 isolate among the SIT53 isolates, which has been rarely reported in the world (Napier et al., 2020).

SIT42 seems to dominate central and south America. In neighboring Guatemala, the most abundant spoligotypes are similar to those found in Mexico: SIT33 (23%), SIT53 (14%), and SIT42 (7%) (Castellanos et al., 2020). In Panama, SIT42 produces 68% of the MDR cases (Realpe et al., 2014; Castellanos et al., 2020) and is also the spoligotype dominant in Colombia (30%) (Rosas et al., 2013). Alternatively, SIT53 in Colombia represents less than 3.4% of the isolates (Rosas et al., 2013).

In Mexico, SIT42 isolates have been found in Monterrey and San Luis Potosi, in the north, at 1.11% and 5.9%, respectively (INEGI, 2015; Flores-Treviño et al., 2015); in Acapulco, in south central Mexico, it produces 5.6% of the cases (Nava-Aguilera et al., 2011); and in Guadalajara, in the west (López-Rocha et al., 2013), 7.4%, compared to 13.7% in Chiapas. This amount is more similar to that found in central and south America.

We found in Chiapas 4.3 lineages to be abundant, particularly sub-lineages 4.3.3 and 4.3.4.2. The Latino-American family (Lineage 4.3) has been observed to predominate in South America (Brynildsrud et al., 2018). Interestingly in a recent study using samples from the Guarani-Kaiowaí indigenous people living in Paraguay and the Brazilian state of Mato Grosso do Sul, Hadi et al. (2021) found that 81% of the isolates belong to sub-lineage 4.3.3, followed by 4.3.4.2 (13%), in a similar way to our findings (Hadi et al., 2021). In both cases, Amerindian people were the hosts, which makes possible the adaptation of these sub-lineages to people with this genetic background, as has been seen in Tanzania where L3.1.1 is highly successful in Dar es Salam (Zweyer et al., 2021). The comparison of our 4.3.3 genomes with those from Hadi et al.’s (2021) study resulted in the formation of a separated cluster with two Amazonian isolates in the group, which strengthen the possible relationship with South American isolates. These Chiapas’ 4.3.3 isolates, even if they were not identical, showed a similar pattern in mutations in genes associated with antimicrobial resistance. When aligned with other 294 lineage 4 isolates, these isolates clustered together with an isolate from Sierra Leona, supporting the African origin of these isolates brought by people enslaved by Portuguese to Brazil. Three more isolates were located close to MTB isolates from Portugal, strengthening this possibility.

The pre-Columbian presence of *M. tuberculosis* has been suggested by the finding of IS6110-positive samples from Peruvian mummies (Klaus et al., 2010). More recently, *M. pinnipedii* DNA has been isolated in South America from human remains, supporting the presence of MTBC infections before the contact with Europeans (Bos et al., 2014). If pre-Columbian *M. tuberculosis* would exist, then it should be demonstrated in Amerindian settlements which have little contact with the rest of the Mexican population. However, we did not find a new lineage or other members of the MTBC complex. Our results support the theory of the origin of TB in Mexico as through contact with Europeans and that 200 years was sufficient time for the organism to spread across the whole country and adapt to the new naïve population.

According to our results, Chiapas 4.3.3 isolates seem to come from Africa *via* Brazil. It seems like from this country this TB lineage spread to the rest of Latin America where it is abundant, including south of Mexico. Interestingly, SIT42 is not predominant in north of Mexico, perhaps due to the recent migration of 4.3.3 to Chiapas or due to differences in host susceptibility to MTB lineages. It is necessary to study the lineages prevailing in the rest of Mexico in order to determine the dissemination and adaptation of the different MTB families.

## Data Availability Statement

The dataset presented in this study can be found in online repositories. The Whole Genome Shotgun project has been deposited at DDBJ/ENA/GenBank under the following accession numbers: M. tuberculosis LIID-CH2 (JAIFJQ000000000), M. tuberculosis LIID-CH3 (JAIFJP000000000), M. tuberculosis LIID-CH6 (JAIFJO000000000), M. tuberculosis LIID-CH14 (JAIFJN000000000), M. tuberculosis LIID-CH15 (JAIFJM000000000), M. tuberculosis LIID-CH17 (JAIFJL000000000), M. tuberculosis LIID-CH18 (JAIFJK000000000), M. tuberculosis LIID-CH19 (JAIFJJ000000000), M. tuberculosis LIID-CH21 (JAIFJI000000000), M. tuberculosis LIID-CH24 (JAIFJH000000000), M. tuberculosis LIID-CH28 (JAIFJG000000000), M. tuberculosis LIID-CH30 (JAIFJF000000000), M. tuberculosis LIID-CH31 (JAIFJE000000000), M. tuberculosis LIID-CH33 (JAIFJD000000000), M. tuberculosis LIID-CH40 (JAIFJC000000000), M. tuberculosis LIID-CH42 (JAIFJB000000000), M. tuberculosis LIID-CH53 (JAIFJA000000000), M. tuberculosis LIID-CH60 (JAIFJZ000000000), M. tuberculosis LIID-CH68 (JAIFJY000000000), M. tuberculosis LIID-CH70 (JAIFJX000000000), M. tuberculosis LIID-CH76 (JAIFJW000000000), M. tuberculosis LIID-CH93 (JAIFJV000000000), M. tuberculosis LIID-CH109 (JAIFJU000000000), and M. tuberculosis LIID-CH117 (JAIFJT000000000). All these sequences belong to BioProject PRJNA751891. The names of the repository/repositories and accession number(s) can be found in the article/supplementary material.

## Ethics Statement

Prior to the start of the study, ethical approval was obtained from the Ecosur Research Ethics Committee (CEI-O-068/14) and from the Ministry of Health for Chiapas (5003/00063) under the project “Mycobacterium tuberculosis spoligotypes from clinical isolates from Chiapas, Mexico.” The microbiological records and basic demographic information for patients were anonymized and de-identified prior to analysis. Informed consent for participation was obtained from patients in a written form, and sputum samples were used only for the purpose of this study.

## Author Contributions

LV-C, FQ, and CM-T contributed to conception and design of the study. JC-G and JO-C organized the database. AG-V, AB-A, HS-P, SM-J, and AR contributed to the collection, culture and identification of clinical samples. LV-C, AA, MS, and PS performed the bioinformative analysis. LV-C wrote the first draft of the manuscript. All authors contributed to manuscript revision, read, and approved the submitted version.

## Funding

This work was supported by PAICYT grant SA152-15 from the Universidad Autónoma de Nuevo León.

## Conflict of Interest

The authors declare that the research was conducted in the absence of any commercial or financial relationships that could be construed as a potential conflict of interest.

The handling editor KKM declared a shared affiliation with the author(s) AA, MS, and PS at the time of review.

## Publisher’s Note

All claims expressed in this article are solely those of the authors and do not necessarily represent those of their affiliated organizations, or those of the publisher, the editors and the reviewers. Any product that may be evaluated in this article, or claim that may be made by its manufacturer, is not guaranteed or endorsed by the publisher.
